# Real-world experience with Janus kinase inhibitors in rheumatoid and psoriatic arthritis: retention and discontinuation factors in a UK six-centre cohort

**DOI:** 10.1093/rap/rkag024

**Published:** 2026-02-17

**Authors:** Joseph Nathan, Bayram Farisogullari, Nicholas Jones, Mohammad Ayoub, James Brown, Sarah Levy, James Brader, Ayse Ersoy, Blossom Israni, Mark Lloyd, Afzal Latheef, Diane Hill, Amybel Taylor, Darshani Arachchige, Hlaing Chitsu, Helen Linklater, Kunal Lather, Luke Gompels, Eman Elfar, Ritu Malaiya, Pedro M Machado, Patrick D W Kiely

**Affiliations:** Department of Rheumatology, St George’s University Hospitals NHS Foundation Trust, London, UK; Department of Neuromuscular Diseases, University College London, London, UK; Department of Rheumatology, Croydon University Hospitals, London, UK; Department of Rheumatology, Croydon University Hospitals, London, UK; Department of Rheumatology, Croydon University Hospitals, London, UK; Department of Rheumatology, Croydon University Hospitals, London, UK; Department of Rheumatology, Frimley Health Foundation Trust, Surrey, UK; Department of Rheumatology, Frimley Health Foundation Trust, Surrey, UK; Department of Rheumatology, Frimley Health Foundation Trust, Surrey, UK; Department of Rheumatology, Frimley Health Foundation Trust, Surrey, UK; Department of Rheumatology, St George’s University Hospitals NHS Foundation Trust, London, UK; Department of Rheumatology, St George’s University Hospitals NHS Foundation Trust, London, UK; Department of Rheumatology, Surrey and Sussex Healthcare NHS Trust, Surrey, UK; Department of Rheumatology, Surrey and Sussex Healthcare NHS Trust, Surrey, UK; Department of Rheumatology, Surrey and Sussex Healthcare NHS Trust, Surrey, UK; Department of Rheumatology, Surrey and Sussex Healthcare NHS Trust, Surrey, UK; Department of Rheumatology, Somerset NHS Foundation Trust, Taunton, UK; Department of Rheumatology, Somerset NHS Foundation Trust, Taunton, UK; Department of Rheumatology, Epsom and St Helier University Hospitals NHS Trust, Surrey, UK; Department of Rheumatology, Epsom and St Helier University Hospitals NHS Trust, Surrey, UK; Department of Neuromuscular Diseases, University College London, London, UK; National Institute for Health Research, University College London Hospitals Biomedical Research Centre, University College London Hospitals NHS Foundation Trust, London, UK; Department of Rheumatology, Northwick Park Hospital, London North West University Healthcare NHS Trust, London, UK; Department of Rheumatology, St George’s University Hospitals NHS Foundation Trust, London, UK; Institute of Medical and Biomedical Education, City St George’s, University of London, London, UK

**Keywords:** rheumatoid arthritis, psoriatic arthritis, Janus kinase inhibitors, retention, real-world data

## Abstract

**Objectives:**

To investigate real-world use of Janus kinase inhibitors (JAKis) prescribed for RA and PsA.

**Methods:**

We conducted a retrospective review of all patients treated with baricitinib, filgotinib, tofacitinib and upadacitinib across six UK rheumatology centres from time of first use in routine care until November 2024. Standardised data were collected and retention was calculated using Kaplan–Meier analysis. Factors associated with JAKi discontinuation were analysed using multivariable Cox regression analyses.

**Results:**

A total of 985 patients [79.5% female, mean age 60 years, RA 849 (86.2%), PsA 136 (13.8%)] were reviewed. MTX and glucocorticoids (GCs) were co-prescribed in 43% and 33.6% of cases, respectively. Among GC users, dose reduction occurred in 18% and complete discontinuation in 61.8%. JAKis were prescribed as a first-line biologic DMARD in 7.5% and as a third-line or later in 78.8%. Median retention was 49 months (range 1–85; 95% CI 42.62, 55.37), with 52.8% remaining on treatment at censure, with no difference between RA and PsA populations. Retention on tofacitinib was significantly lower than baricitinib (*P* < 0.001) and upadacitinib (*P* = 0.007). Variables significantly associated with higher JAKi discontinuation risk were female sex [hazard ratio (HR) 1.66 (95% CI 1.28, 2.16)], increasing age [HR 1.01 (95% CI 1.00, 1.02)] and tofacitinib (*vs* baricitinib) treatment [HR 2.27 (95% CI 1.64, 3.14)]. Among RA patients, MTX co-prescription was associated with a lower discontinuation risk [HR 0.81 (95% CI 0.66, 0.98)].

**Conclusion:**

In a real-world setting, JAKi demonstrated high retention in RA and PsA, with a GC-sparing effect. Retention is lower for female sex, older age, tofacitinib *vs* baricitinib and use without MTX in RA, whereas place in the treatment pathway did not impact retention outcomes.

Key messages

- Janus kinase inhibitor retention is high in real-world practice with a glucocorticoid-sparing effect.
- Retention is significantly lower with tofacitinib *vs* baricitinib, increasing age, female sex and without MTX.
- Retention is not influenced by place in the biologic treatment pathway.

## Introduction

Janus kinase inhibitors (JAKis) have been licensed in the UK to treat RA and PsA since 2017, following randomized controlled phase III trials demonstrating their efficacy and acceptable toxicity in patients both naïve and previously treated with biologic DMARDs (bDMARDs) [1]. Subsequently, the findings of the ORAL Surveillance Study have raised long-term safety concerns of JAKis in relation to TNF-α inhibitors in RA patients [2], generating uncertainties about their use in clinical practice [3]. An expert consensus statement has provided ‘points to consider’ once the decision to use a JAKi has been made but has been unable to provide advice on when a JAKi should be used in treatment pathways, either in general or on an individual person basis [4], leaving this decision to the individual practitioner. In the UK, the number of people prescribed JAKi for any indication increased by 358% between January 2019 and May 2024 [5]. Despite this widespread uptake, their utility in routine care has not been well described and real-world evidence regarding efficacy, safety and retention is limited.

Our aim was to assess contemporary patterns of use of JAKis in a large multicentre retrospective series of patients with rheumatic diseases since they were first introduced into routine care. We report demographic features, the place used in the RA and PsA treatment pathway, overall retention, comparative retention between each JAKi, factors associated with retention, reasons for discontinuation and adverse events of special interest.

## Methods

### Study design and population

This retrospective observational real-world study utilised data from all patients diagnosed with RA and PsA who received any JAKi since they were first available in routine care (2017) through censure in October 2024 in six secondary care rheumatology centres in South London, Surrey and Somerset, UK. The diagnoses were established by the treating physicians and treatment decisions reflected routine care, with no patients involved in a therapeutic clinical trial or other fixed algorithm of care. All current and past JAKi-treated patients who had received at least one dose of any JAKi were included.

### Data collection and measures

Data collection was standardised across all centres and included age, sex, disease indication (RA, PsA), JAKi agent (baricitinib, filgotinib, tofacitinib, upadacitinib), duration of treatment, sequence of JAKi in the bDMARD/targeted synthetic DMARD (tsDMARD) treatment pathway (first, second, third, fourth line or later), MTX and glucocorticoid (GC) co-prescription, dose reduction and discontinuation. Reasons for JAKi discontinuation were categorised as either ineffectiveness (primary or secondary failure, as determined by the rheumatologist), adverse events or concerns specific to the findings of the ORAL Surveillance Study [2]. Adverse events leading to discontinuation, including venous thromboembolic events (VTEs), major cardiovascular events and new or recurrent malignancy were also recorded. Variables were collected retrospectively from hospital records review at the time of censure in late 2024.

### Assessment of retention rate

Analysis was conducted on demographics, clinical characteristics and treatment features for all patients and stratified by disease (RA, PsA) and JAKi (baricitinib, filgotinib, tofacitinib, upadacitinib) subtypes. Comparative analysis was conducted categorizing patients into two groups based on their treatment situation at the time of data collection: those who continued *vs* those who discontinued JAKi therapy. The duration of JAKi use was defined as the number of months from treatment initiation to either discontinuation or the most recent follow-up while on treatment.

### Statistical analysis

Statistical analysis was performed using SPSS Statistics for Windows version 28.0 (IBM, Armonk, NY, USA). Categorical variables are presented as frequency distributions. Continuous variables are summarised using the mean and s.d. Comparisons of numerical variables between groups were performed using the independent samples *t*-test, while categorical variables were compared using the chi-squared test. If the assumptions of the chi-squared test were not met, Fisher’s exact test was applied. Time-to-event analyses for JAKi retention were conducted using the Kaplan–Meier method, with between-group comparisons assessed using the logrank test. *P*-values <0.05 were considered to be statistically significant.

Factors associated with the discontinuation of JAKi were assessed using univariable and multivariable Cox regression analyses and reported as hazard ratios (HRs) with 95% CIs. The following covariates were included in the models: age, sex, rheumatic disease diagnosis [RA (reference) and PsA], sequence of JAKi use among biologic treatments [first line (reference), second line, third line and fourth line or more), type of JAKi [baricitinib (reference), filgotinib, tofacitinib and upadacitinib), MTX co-prescription at JAKi initiation and GC co-prescription at JAKi initiation.

### Ethical approval and consent

Retrospective case note review did not require ethical approval or consent according to local audit/service evaluation governance policies. All patient data were pseudo-anonymised. The study complied with the principles of the Declaration of Helsinki and local data protection regulations.

## Results

### Demographics

**Figure 1:**
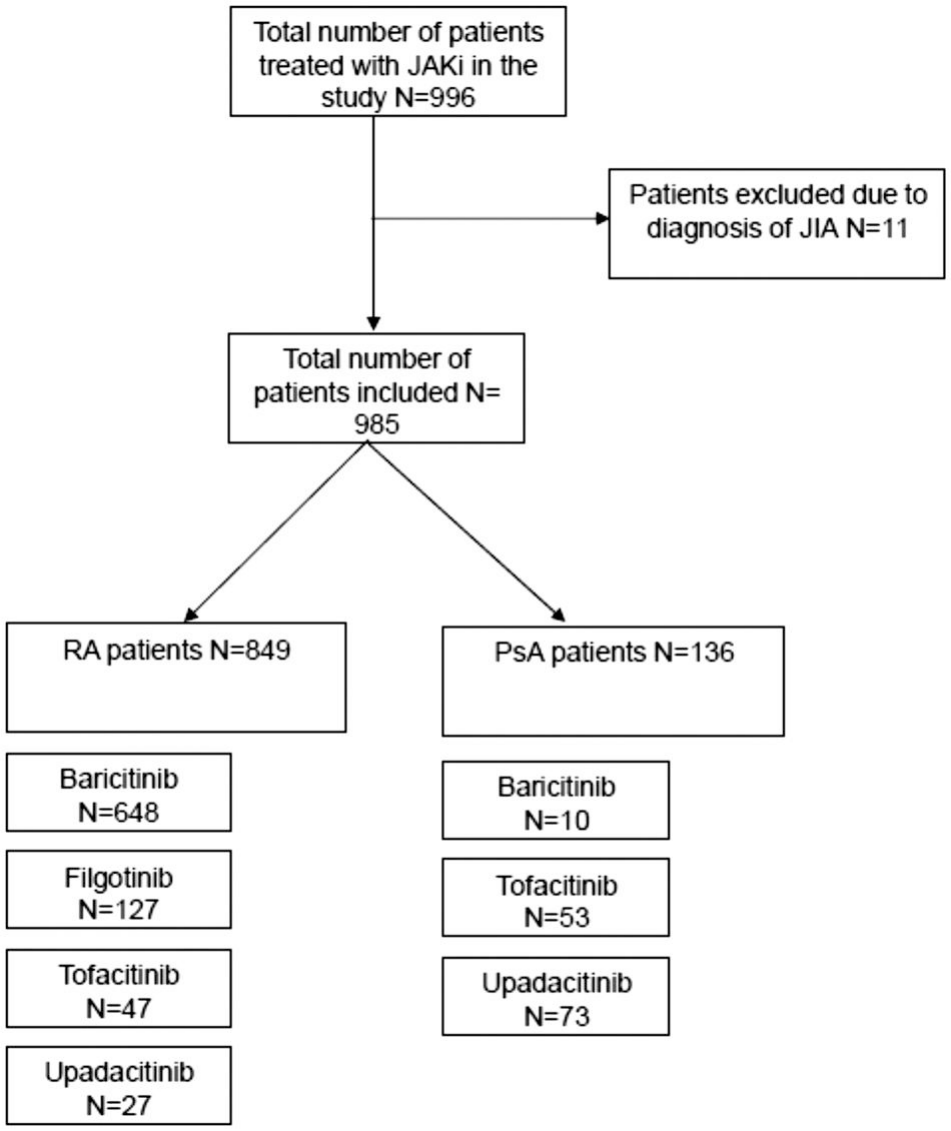
Patient inclusion with subgroups by RA and PsA and individual JAKi prescribed

Data concerning 996 patients were collected, including 849 with RA, 136 with PsA and 11 with JIA. No further analysis of patients with JIA was performed given the low numbers, leaving 985 patients with either RA or PsA, with numbers prescribed each JAKi shown in Fig. 1. The total patient-years exposure to JAKi was 2447.25 years. Summary frequencies of demographic characteristics, JAKi prescribed, sequence of JAKi treatment in the biologic pathway, co-prescription with MTX and GC, dose reduction or discontinuation of these and reasons for JAKi discontinuation are shown in Table 1. The majority of JAKi use was as third or fourth line or later (78.8%) in the biologic pathway compared with first-line use in 7.5%. MTX was co-prescribed in 43.4% of cases and subsequently discontinued in 5.9%.

**Table 1:** Demographic and clinical characteristics of the study population.

Characteristics	Values
Age, years, mean (s.d.)	60.1 (12.8)
Female, *n* (%)	783 (79.5)
Diagnosis, *n* (%)	
RA	849 (86.2)
PsA	136 (13.8)
Type of JAKi, *n* (%)	
Baricitinib	658 (66.8)
Filgotinib	127 (12.9)
Tofacitinib	100 (10.2)
Upadacitinib	100 (10.2)
Sequence of JAKi treatment (*n* = 984), *n* (%)	
First line	74 (7.5)
Second line	135 (13.7)
Third line	285 (29)
Fourth line or later	490 (49.8)
MTX use at initiation of JAKi treatment, *n* (%)	427 (43.4)
MTX discontinuation during JAKi treatment (*n* = 427), *n* (%)	25 (5.9)
Corticosteroid use at initiation of JAKi treatment (*n* = 983), *n* (%)	331 (33.6)
Corticosteroid dose reduction or discontinuation during JAKi treatment (*n* = 327), *n* (%)	
Dose reduction	59 (18)
Discontinuation	202 (61.8)
Reason for discontinuation, *n* (%)	
All reasons	457 (46.4)
Primary inefficacy	97 (9.8)
Secondary inefficacy	116 (11.8)
Adverse effects	182 (18.5)
Oral surveillance scare	62 (6.3)

*N* = 985 unless stated otherwise.

GCs were co-prescribed at JAKi initiation in 33.6% of patients (331/983). Among 327 patients with complete GC follow-up data, the GC dose was reduced in 18% and GCs were completely discontinued in 61.8%.

### Retention, whole population and disease specific

**Figure 2:**
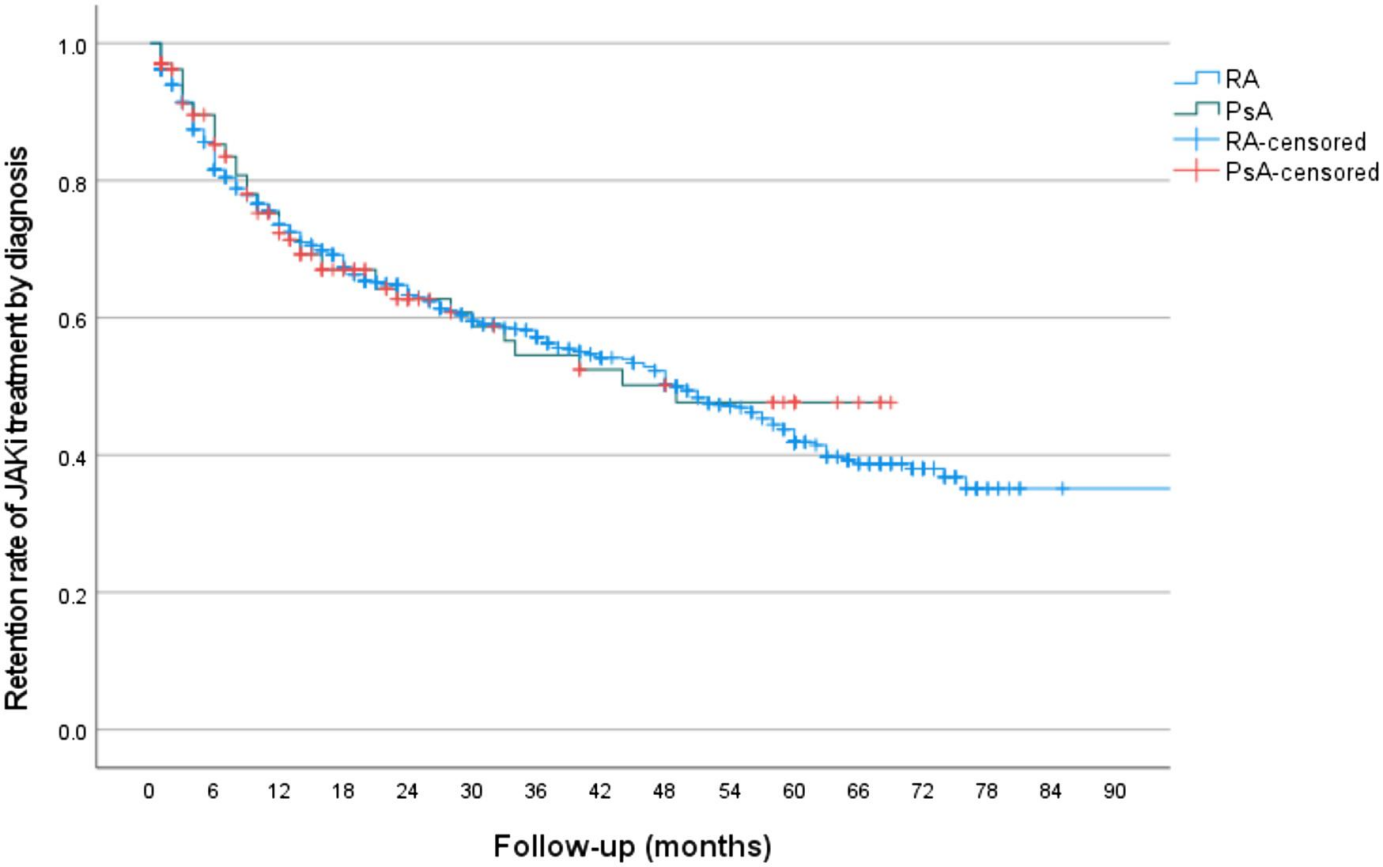
Kaplan–Meier retention curve for patients with RA and PsA treated with any JAKi (*P* = 0.719).

Of 984 patients treated with any JAKi for either RA or PsA, 520 (52.8%) remained on treatment at the time of data collection, with a median survival of 49 months (range 1–85; 95% CI 42.62, 55.37) (Supplementary Fig. S1). The retention rate for RA was 50.9% and for PsA was 65.2%, with a median survival of 50 and 49 months, respectively; a non-significant difference (Fig. 2).

### Retention: individual JAKi whole population and disease specific

**Figure 3:**
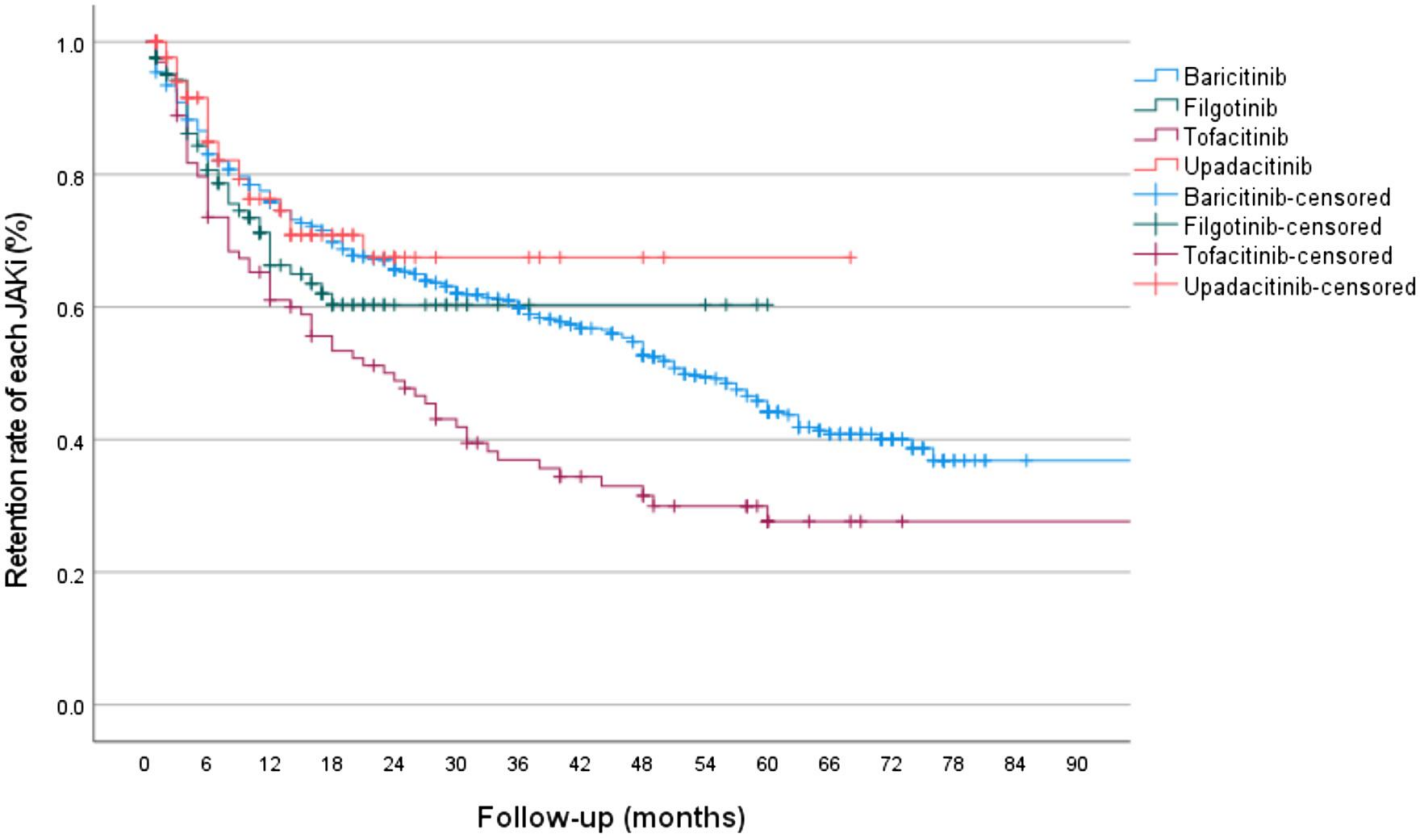
Kaplan–Meier retention curve for all patients treated with individual JAKis. Baricitinib *vs* tofacitinib (*P* < 0.001) and upadacitinib *vs* tofacitinib (*P* = 0.007).

Retention for individual JAKis is shown for RA and PsA patients combined in Fig. 3. The median survival on baricitinib was 52 months (95% CI 46.9. 57.1), 39 months (95% CI 34.1, 44.5) for filgotinib, 24 months (95% CI 14.3, 33.7) for tofacitinib and 49 months (95% CI 41.8, 55.7) for upadacitinib (overall *P* < 0.001), with 48.6%, 69.3%, 34.3% and 78.0%, respectively, remaining on treatment at censure. This was significantly different between baricitinib and tofacitinib (*P* < 0.001) and upadacitinib and tofacitinib (*P* = 0.007). Similar patterns of retention for individual JAKis were seen in the RA and PsA populations separately (Supplementary Figs S2 and S3).

### Predictors of retention: whole population, disease specific and individual JAKis

Univariable and multivariable Cox regression analyses of variables influencing JAKi retention (all JAKis combined) for all patients are shown in Supplementary Table S1 and Table 2, respectively. Multivariable analysis for the whole population showed a significantly higher relative risk of JAKi discontinuation for female sex [HR 1.66 (95% CI 1.28, 2.16)], increasing age [HR 1.01 (95% CI 1.0, 1.02)] and treatment with tofacitinib compared with baricitinib [HR 2.27 (95% CI 1.64, 3.14)]. In contrast, in RA *vs* PsA, first-line biologic pathway position *vs* second-line or later and co-prescription with MTX or GC were not associated with JAKi retention. However, for the RA patients alone, co-prescription with MTX had a significantly lower risk of JAKi discontinuation [HR 0.81 (95% CI 0.66, 0.98)], but not for PsA (Table 2).

**Table 2:** Multivariable Cox regression analyses of variables influencing discontinuation of all JAKis: combined dataset and for RA and PsA separately.

Variables	All patients (*n* = 982)		RA patients (*n* = 847)		PsA patients (*n* = 135)	
	HR (95% CI)	*P*-value	HR (95% CI)	*P*-value	HR (95% CI)	*P*-value
Age (years)	1.01 (1.00, 1.02)	0.044	1.01 (1.00, 1.02)	0.061	1.01 (0.98, 1.03)	0.53
Gender						
Male	Reference		Reference		Reference	
Female	1.66 (1.28, 2.16)	<0.001	1.62 (1.22, 2.15)	<0.001	1.63 (0.82, 3.23)	0.16
Diagnosis			NA	NA	NA	NA
RA	Reference					
PsA	0.70 (0.47, 1.05)	0.083				
Order of JAKi						
First	Reference		Reference		Reference	
Second or more	1.05 (0.73, 1.51)	0.79	1.09 (0.75, 1.57)	0.67	0.04 (0.004, 0.39)	0.006
Type of JAKi						0.43
Baricitinib	Reference		Reference		Reference	
Filgotinib	1.19 (0.85, 1.67)	0.31	1.20 (0.85, 1.69)	0.29		0.40
Tofacitinib	2.27 (1.64, 3.14)	<0.001	2.40 (1.69, 3.40)	<0.001	1.60 (0.54, 4.80)	0.90
Upadacitinib	1.22 (0.74, 2.02)	0.43	1.03 (0.48, 2.18)	0.95	1.08 (0.33, 3.50)	
MTX co-prescription when JAKi started (yes *vs* no)	0.84 (0.70, 1.01)	0.066	0.81 (0.66, 0.98)	0.032	1.24 (0.66, 2.34)	0.51
Steroid co-prescription when JAKi started (yes *vs* no)	1.11 (0.91, 1.34)	0.32	1.10 (0.90, 1.35)	0.36	1.10 (0.55, 2.20)	0.78

For the individual JAKi, discontinuation was also more likely for female *vs* male sex for baricitinib and tofacitinib in the whole population, with no significant effect of other variables on retention, including place in the treatment pathway, evident for each JAKi separately (Table 3).

**Table 3:** Multivariable Cox regression analyses of variables influencing discontinuation of individual JAKis: combined RA and PsA dataset.

Variables	Baricitinib (*n* = 657)		Filgotinib (*n* = 127)		Tofacitinib (*n* = 98)		Upadacitinib (*n* = 86)	
	HR (95% CI)	*P*-value	HR (95% CI)	*P*-value	HR (95% CI)	*P*-value	HR (95% CI)	*P*-value
Age (years)	1.01 (0.99, 1.01)	0.27	1.02 (0.99, 1.05)	0.11	1.02 (0.99, 1.05)	0.078	1.03 (0.99, 1.07)	0.17
Gender								
Male	Reference		Reference		Reference		Reference	
Female	1.56 (1.14, 2.12)	0.005	1.23 (0.47, 3.17)	0.67	2.52 (1.24, 5.14)	0.011	1.36 (0.51, 3.60)	0.54
Diagnosis			NA	NA				
RA	Reference				Reference		Reference	
PsA	0.77 (0.29, 2.08)	0.61			0.68 (0.39, 1.16)	0.15	0.71 (0.28, 1.85)	0.49
Order of JAKi								
First	Reference		Reference		Reference		Reference	
Second or more	0.82 (0.55, 1.22)	0.33	1.82 (0.54, 6.11)	0.33	2.05 (0.60, 6.95)	0.25	Unreliable HR due to highly imbalanced data	0.98
Type of JAKi								
Baricitinib	NA	NA	NA	NA	NA	NA	NA	NA
Filgotinib								
Tofacitinib								
Upadacitinib								
MTX co-prescription when JAKi started (yes *vs* no)	0.85 (0.68, 1.05)	0.14	0.97 (0.50, 1.86)	0.92	0.69 (0.41, 1.17)	0.17	0.69 (0.25, 1.97)	0.49
Steroid co-prescription when JAKi started (yes *vs* no)	1.09 (0.88, 1.37)	0.42	1.14 (0.57, 2.26)	0.71	1.17 (0.68, 2.01)	0.58	0.65 (0.21, 1.97)	0.44

### Reasons for discontinuation: whole population, disease specific and individual JAKis

JAKis were discontinued in 46.4% of the whole population, with adverse events leading to 18.5% stopping treatment and 6.3% pre-emptively stopping specifically due to the findings of the ORAL Surveillance study (Table 1). The reasons for discontinuation in the RA and PsA populations separately are shown in Supplementary Table S2, with no significant differences between these disease groups for all JAKis, nor for each individual JAKi (Supplementary Table S3A–D).

Adverse events were in line with those associated with JAKis and led to treatment discontinuation due to recurring infections in 40 (4%) patients. VTEs occurred in 14 (1.4%) patients; 10 pulmonary embolism (9 baricitinib, 1 filgotinib) and 4 deep vein thrombosis (all baricitinib) events, with a mean time to event of 29 months (range 6–75). The VTE event rate was 0.57/100 patient-years. Other adverse events included cerebrovascular events in 12 (1.2%; 0.49/100 patient-years), cardiac events in 7 (0.7%; 0.29/100 patient-years) and cancer in 9 (0.9%; 0.37/100 patient-years).

## Discussion

We found a high JAKi treatment retention rate of 52.8% (median survival 49 months) in a large series of 985 patients with RA and PsA treated with JAKi according to usual practice in six UK centres. This real-world evidence includes all patients ever treated with a JAKi for a rheumatic disease dating since these became available for routine care. In nearly 80% of cases JAKis were used third or fourth line or later in the advanced therapies treatment pathway and only rarely before any bDMARDs (7.5%). Treatment survival compares very favourably to TNF inhibitors given to biologic-experienced RA patients, where retention at 4 years is ≈40% [6], and to biologic-naïve patients treated without MTX, where 4-year retention rates are 40% for adalimumab and etanercept combined in a Canadian cohort [7]. Similarly, in the Swiss RA register [8] and the JAK-pot collaboration of 19 registers, retention is equivalent across all modes of action of bDMARDs and JAKis [9].

While GCs were co-prescribed in 33.6% of patients at JAKi initiation, dose reduction or complete GC withdrawal occurred in 80% of these patients during follow-up. This provides good evidence that JAKis are effective steroid-sparing therapies for RA and PsA in real-world practice. Although we are unable to report the dose or duration of prednisolone treatment in individual cases, we interpret the finding that such a high number of cases were able to reduce the dose or discontinue prednisolone as a surrogate of JAKi efficacy in patients with established disease using these therapies late in the treatment pathway. In the Japanese ANSWER cohort, patients taking prednisolone at doses of ≥5 mg had a higher risk of JAKi discontinuation due to adverse events [10], but this was not replicated in our series, perhaps because of the high rate of GC dose reduction and discontinuation.

Retention on JAKi was not different between RA and PsA, and factors associated with discontinuation were female sex and increasing age in the combined cohort, and no co-prescription of MTX in the RA population but not the PsA population. The lower retention with increasing age and female *vs* male patients concurs with findings from the ANSWER cohort [10]. Similarly in the JAK-pot collaboration, increasing age and female patients were at higher risk of discontinuation due to adverse events [9].

The lower retention of tofacitinib compared with baricitinib, overall 34.3% *vs* 48.6%, respectively, cannot be explained by a single reason, as the relative proportions of patients discontinuing due to primary and secondary inefficacy, adverse events and following the ORAL Surveillance study were comparable. A differential reason for discontinuation between these therapies may be lost because the overall number treated with tofacitinib was lower than for baricitinib. Furthermore, while the significantly higher risk of discontinuation of tofacitinib compared with baricitinib might suggest differences in efficacy, this cannot be evaluated, as disease activity data were not collected. The higher retention rate of baricitinib compared with tofacitinib in routine care is consistent with findings from the Japanese FIRST registry [11], where baricitinib was associated with a higher rate of Clinical Disease Activity Index remission. Similarly, a network meta-analysis of comparative efficacy in four randomized controlled trials found baricitinib had a greater probability than tofacitinib of achieving a 20% or 50% improvement in ACR criteria in RA patients [12]. In contrast the ANSWER cohort found no significant differences in retention rates between baricitinib and tofacitinib, either due to lack of efficacy or adverse events, suggesting variability across different datasets [10].

The lack of an influence of the place in the biologic treatment pathway on combined JAKi retention concurs with the ANSWER cohort [10, 13], an Italian cohort of tofacitinib-treated RA patients [14] and baricitinib-treated RA patients in the FIRST registry [11]. In contrast, bDMARD treatment before tofacitinib was associated with treatment resistance in RA patients in the FIRST registry [11], and in a series from Spain, previous failure of bDMARDs was associated with poor response to JAKi in RA but not PsA patients [15]. The apparent consistency in JAKi efficacy across all stages of the b/tsDMARD treatment pathway contrasts sharply with bDMARDs, where efficacy tends to be highest when used as first-line therapy [6, 13].

In the ANSWER cohort, prior ineffectiveness of an anti-IL-6 receptor antibody increased the risk of subsequent JAKi discontinuation due to ineffectiveness [10]. We were unable to assess JAKi retention in relation to specific prior bDMARD treatments, but subanalysis in a subset of 67 cases confirmed prescribing practice in line with EULAR recommendations [16, 17]. Thus the majority of patients were treated with a TNF inhibitor as a first-line bDMARD, and second-/third-line bDMARD choices were generally a second TNF inhibitor followed by rituximab or tocilizumab (RA) or an IL-17 inhibitor (PsA) prior to use of a JAKi.

Although not mandatorily licensed for use with MTX, nearly half of the cohort were co-prescribed this drug at JAKi initiation, and this was subsequently discontinued in only 5.9%. The low MTX discontinuation rate may reflect caution among prescribers given the positioning of JAKi late in the treatment pathway after several previous bDMARDs had failed. The significantly lower risk of JAKi discontinuation in RA patients who were co-prescribed MTX, but no influence on retention in PsA patients, may be an important clinical finding, similar to TNF inhibitors [7]. However, this needs replication, as the co-prescription of MTX in baricitinib- and tofacitinib-treated RA patients in the ANSWER cohort did not significantly affect drug retention [10].

The incidence of VTE, at 0.57/100 patient-years, was lower than the rate recently reported from the Swedish Rheumatology Quality Register, where the event rate for JAKi was double that, at 1.13/100 patient-years [18]. The exposure period to JAKi in the Swedish data was nearly double ours, at 4184 *vs* 2447 patient-years. Perhaps with longer exposure an exponentially greater number of VTE events may occur, as illustrated by the time course of VTE, with JAKi exceeding biologic agents only after 2 years [18]. Like the Swedish data, we also observed more pulmonary embolism than deep vein thrombosis, although the reasons for this remain unclear [18]. We did not collect comprehensive co-morbidity data to enable assessment of specific risk factors for these events.

The strengths of our findings come from the large patient numbers treated across six secondary care centres over a period of up to 85 months, without protocol or external control on treatment decisions. Thus we can convey the way JAKis have been used in UK practice since first introduced for routine care. The limitations reflect the different years that individual JAKis were licensed, such that these data are not balanced with more RA (86.2%) than PsA (13.8%) patients and more patients receiving baricitinib (66.8%) compared with filgotinib (12.9%), tofacitinib and upadacitinib (10.2% each). Other limitations of real-world evidence are a lack of standardised criteria for diagnosis, potential channelling bias with respect to the use of therapies, both biologic and other anti-rheumatic drugs, and treatment escalation and switch decisions at the discretion of each rheumatologist. We did not collect disease activity data or patient-reported outcome measures, instead using retention as a surrogate of efficacy, and have not focused on co-morbidities and other factors contributing to adverse events. While we observe an association between MTX use and higher JAKi retention in RA patients, as well as a general GC-sparing effect, we do not have data concerning doses or duration of treatment for either. Finally, we did not include RA/PsA disease duration in the analyses. This is unlikely to be a confounder, as later sequencing of JAKi treatment in the biologic treatment pathway did not influence retention.

In summary, in nearly 1000 patients treated with JAKi for RA and PsA across six UK centres, we found high retention rates. Factors associated with higher risk of discontinuation included female sex, increasing age, baricitinib *vs* tofacitinib treatment and, in RA patients, use without MTX. JAKis were more frequently used as third-line or later therapy, although treatment placement had no impact on retention. A GC-sparing effect was evident and adverse events were in line with this class of immune suppressive therapies, including VTE, with pulmonary embolism occurring more frequently than deep vein thrombosis.

## Supplementary Material

rkag024_Supplementary_Data

## Data Availability

Data sharing requests can be submitted after 1 year following publication of the study results to the corresponding author, who will provide a data access request form. Data sharing requests will be considered by the authors on a case-by-case basis and data will be shared if the request is considered reasonable, of scientific interest and legally and ethically possible.
