# Supplementary material for: Real-world experience with Janus kinase inhibitors in rheumatoid and psoriatic arthritis: retention and discontinuation factors in a UK six-centre cohort
Source: Rheumatol Adv Pract. 2026 Feb 17;10(2):rkag024. doi: 10.1093/rap/rkag024 (PMC13038248; doi:10.1093/rap/rkag024)
Supplement: rkag024_Supplementary_Data [file rkag024_supplementary_data.zip › JAKi_retention_RWE_manuscript,_Supplementary_materials_v3.docx]

**Supplementary Figure S1.**

Kaplan-Meier retention curve for all patients treated with any JAK inhibitor


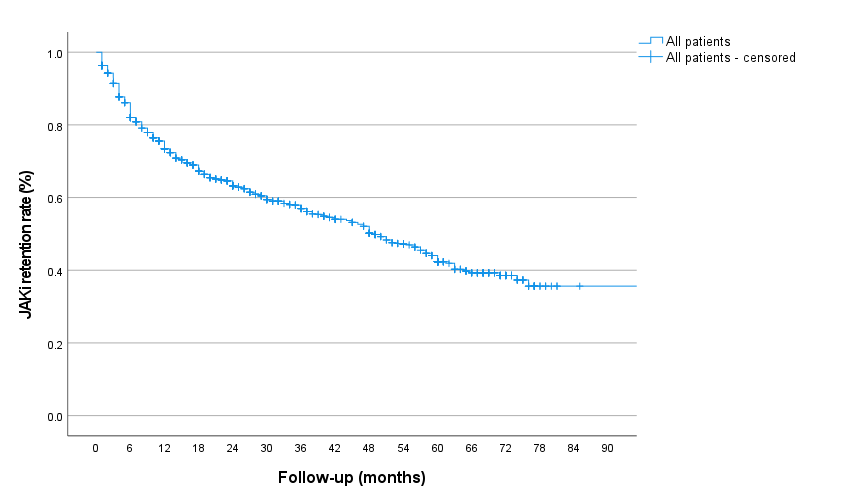


**Supplementary Figure S2**

Kaplan-Meier retention curve for Rheumatoid arthritis (RA) patients treated with individual JAK inhibitors


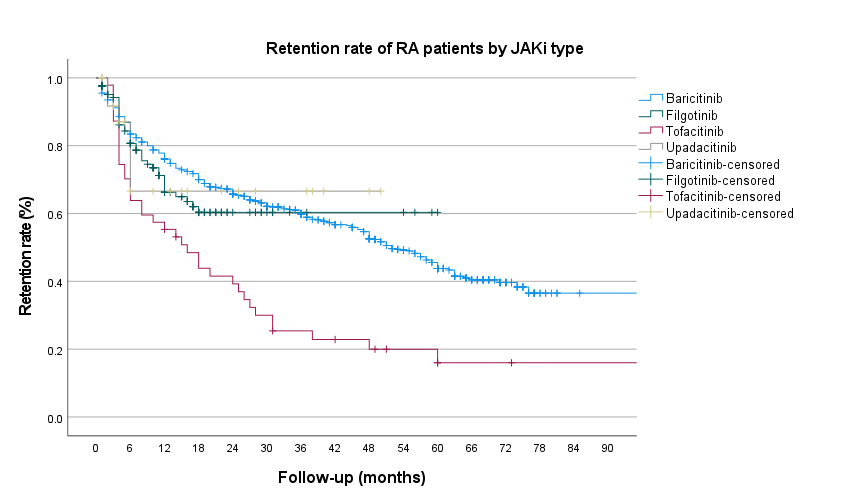


**Supplementary Figure S3**

Kaplan-Meier retention curve for Psoriatic arthritis (PsA) patients treated with individual JAK inhibitors

**
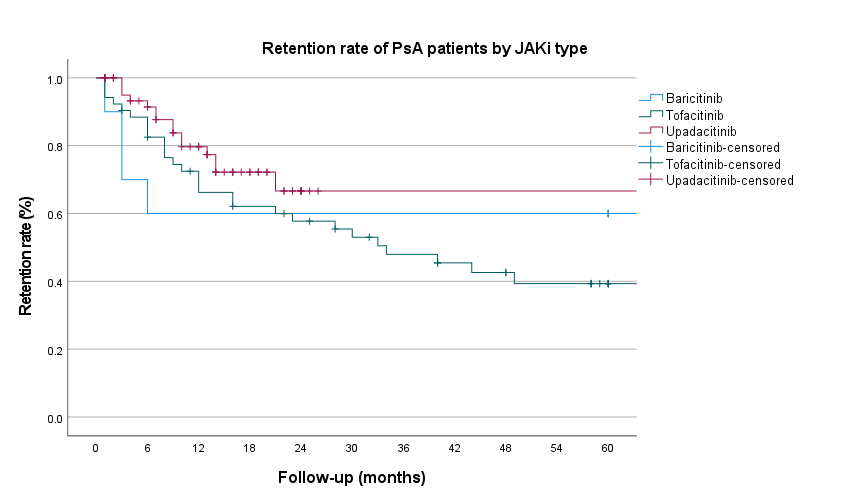
**

**Supplementary Table S1** Univariable Cox regression analyses of variables influencing discontinuation of all JAKi: combined RA and PsA dataset

| **Univariable Cox regression analysis for all patients** | | | |
| --- | --- | --- | --- |
| **Variables** | **Number of patients** | **HR (95% CI)** | **p-value** |
| **Age** | 984 | 1.01 (0.99-1.01) | 0.072 |
| **Gender** (female vs male) | **984** | **1.61 (1.25-2.09)** | **<0.001** |
| **Diagnosis**  **RA**  **PsA** | 984 | **Reference**  0.95 (0.70-1.28) | 0.72 |
| **Order of JAKi**  **1 ^st^**  **2^nd^ or more** | 983 | **Reference**  0.99 (0.69-1.42) | 0.97 |
| **Type of JAKi**  **Baricitinib**  **Filgotinib**  **Tofacitinib**  **Upadacitinib** | 984 | **Reference**  1.18 (0.84-1.65)  **1.65 (1.27-2.16)**  0.87 (0.56-1.34) | **0.02**  0.35  **<0.001**  0.52 |
| **MTX co-prescription when JAKi started (yes vs no)** | 984 | 0.86 (0.72-1.03) | 0.11 |
| **MTX stopped (yes vs no)** | 427 | 1.03 (0.57-1.84) | 0.94 |
| **Steroid co-prescription when JAKi started (yes vs no)** | 983 | 1.17 (0.97-1.42) | 0.10 |
| JAKi: Janus kinase inhibitors; PsA: Psoriatic arthritis; RA: Rheumatoid arthritis; MTX: methotrexate | | | |

**Supplementary Table S2**

Reasons for discontinuing any JAKi, total population and disease sub-groups; p=0.113

|  | Primary Inefficacy  N (%) | Secondary Inefficacy  N (%) | Adverse Effects  N (%) | Oral Surveillance Scare  N (%) | Total  N |
| --- | --- | --- | --- | --- | --- |
| RA | 84 (20.5) | 103 (25.2) | 170 (41.6) | 52 (12.7) | 409 |
| PsA | 13 (27.1) | 13 (27.1) | 12 (25) | 10 (20.8) | 48 |
| Total population | 97 (21.2) | 116 (25.4) | 182 (39.8) | 62 (13.6) | 457 |
| JAKi: Janus kinase inhibitors; RA: Rheumatoid Arthritis; PsA: Psoriatic Arthritis. | | | | | |

**Supplementary Table S3A.** Reasons for discontinuing Baricitinib, total population and disease sub-groups; p=0.3

|  | Primary Inefficacy  N (%) | Secondary Inefficacy  N (%) | Adverse Effects  N (%) | Oral Surveillance Scare  N (%) | Total  N |
| --- | --- | --- | --- | --- | --- |
| RA | 62 (19) | 92 (28.1) | 133 (40.7) | 40 (12.2) | 327 |
| PsA | 2 (50) | 0 | 2 (50) | 10 | 4 |
| Total population | 64 (19.3) | 92 (27.8) | 135 (40.8) | 40 (12.1) | 331 |
| RA: Rheumatoid Arthritis; PsA: Psoriatic Arthritis. | | | | | |

**Supplementary Table S3B.** Reasons for discontinuing Filgotinib, Rheumatoid Arthritis.

|  | Primary Inefficacy  N (%) | Secondary Inefficacy  N (%) | Adverse Effects  N (%) | Oral Surveillance Scare  N (%) | Total  N |
| --- | --- | --- | --- | --- | --- |
| RA | 13 (33.3) | 5 (12.8) | 17 (43.6) | 4 (10.3) | 39 |
| RA: Rheumatoid Arthritis. | | | | | |

**Supplementary Table S3C.** Reasons for discontinuing Tofacitinib, total population and disease sub-groups; p=0.16

|  | Primary Inefficacy  N (%) | Secondary Inefficacy  N (%) | Adverse Effects  N (%) | Oral Surveillance Scare  N (%) | Total  N |
| --- | --- | --- | --- | --- | --- |
| RA | 8 (22.2) | 5 (13.9) | 18 (50) | 5 (13.9) | 36 |
| PsA | 7 (25) | 9 (32.1) | 7 (25) | 5 (17.9) | 28 |
| Total population | 15 (23.4) | 14 (21.9) | 25 (39.1) | 10 (15.6) | 64 |
| RA: Rheumatoid Arthritis; PsA: Psoriatic Arthritis. | | | | | |

**Supplementary Table S3D.** Reasons for discontinuing Upadacitinib, total population and disease sub-groups; p=0.82

|  | Primary Inefficacy  N (%) | Secondary Inefficacy  N (%) | Adverse Effects  N (%) | Oral Surveillance Scare  N (%) | Total  N |
| --- | --- | --- | --- | --- | --- |
| RA | 1 (14.3) | 1 (14.3) | 2 (28.6) | 3 (42.9) | 7 |
| PsA | 4 (25) | 4 (25) | 3 (18.8) | 5 (31.3) | 16 |
| Total population | 5 (21.7) | 5 (21.7) | 5 (21.7) | 8 (34.8) | 23 |
| RA: Rheumatoid Arthritis; PsA: Psoriatic Arthritis. | | | | | |

|  |
| --- |
